# Genetics of irritable bowel syndrome

**DOI:** 10.1186/s40348-016-0038-6

**Published:** 2016-02-12

**Authors:** Maria Henström, Mauro D’Amato

**Affiliations:** Department of Biosciences and Nutrition, Karolinska Institutet, Stockholm, Sweden; Department of Medicine Solna, Karolinska Institutet, Stockholm, Sweden; BioCruces Health Research Institute and Ikerbasque, Basque Foundation for Science, Bilbao, Spain

**Keywords:** Genetics, Genome-wide association study, Irritable bowel syndrome

## Abstract

Irritable bowel syndrome (IBS) is a common condition with a complex and largely unknown etiology. There is no cure, and treatment options are mainly directed to the amelioration of symptoms. IBS causes reduced quality of life and poses considerable repercussions on health and socioeconomic systems. There is a heritable component in IBS, and genetic research is a valuable tool for the identification of causative pathways, which will provide important insight into the pathophysiology. However, although some gene-hunting efforts have been conducted and a few risk genes proposed, IBS genetic research is lagging behind compared to other complex diseases. In this mini-review, we briefly summarize existing genetic studies, discuss the main challenges in IBS genetic research, and propose strategies to overcome these challenges for IBS gene discovery.

## Irritable bowel syndrome prevalence

Irritable bowel syndrome (IBS) is the most commonly diagnosed functional gastrointestinal disorder (FGID) with a worldwide prevalence of 10–20 %, predominantly among women [1]. Its clinical appearance varies but is usually characterized by recurrent abdominal pain or discomfort accompanied by changes in bowel habits and diarrhea (IBS-D), constipation (IBS-C), or both (mixed, IBS-M). In the absence of reliable biomarkers or specific laboratory tests, consensus criteria towards a positive diagnosis have been developed: the symptom-based Rome III criteria [2]. Due to a complex and not fully elucidated pathophysiology, there is no cure, and available treatment options can only be directed to amelioration of symptoms in a trial-and-error approach based on patients’ individual symptomatology. Comorbidity with other FGIDs as well as psychological conditions such as depression and anxiety is observed, as well as certain non-gastrointestinal non-psychological comorbidities including fibromyalgia, chronic fatigue syndrome, and chronic pelvic pain [3]. IBS negatively affects quality of life, which reflects in increased work and school absenteeism, decreased work productivity, and higher utilization of the health care system, with considerable repercussions on health and socioeconomic systems [3, 4].

## IBS pathophysiology

**Fig. 1:**
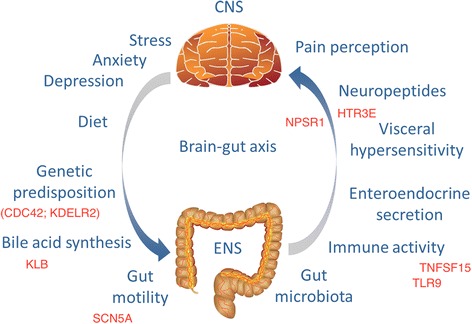
Factors proposed to be involved in the complex pathophysiology of irritable bowel syndrome. *CNS* central nervous system, *ENS* enteric nervous system. Prominent, proposed IBS-risk genes from Table 1 are also reported, by positioning them based on the (most likely) mechanistic involvement in the mentioned pathways

Structural abnormalities, tissue damage, or other organic explanations are typically absent in IBS. Therefore, this condition is traditionally classified as a functional gastrointestinal disorder. Due to significant associations with anxiety, depression, and other psychiatric conditions, IBS has often been considered to be a psychosomatic disorder. However, more recent research has contributed to the elucidation of several mechanisms that may play important roles in IBS [5–7], see Fig. 1. We now understand that mucosal immune activation, inflammatory cells, and elevated inflammatory markers may be present in IBS, at least in a subset of patients. Prior gastroenteritis (post-infectious IBS; PI-IBS), described effects of antibiotics and probiotics on IBS symptoms, and the observation of different gut microbiota profiles in patients and controls all speak for an important role of gut flora in IBS [7]. In addition, diet is recognized in some patients as an important trigger of gut symptoms, and dietary therapeutic approaches have been recently developed, such as the low-FODMAP diet where poorly absorbed and rapidly fermentable carbohydrates are avoided [8]. Visceral hypersensitivity and altered pain perception also appear to be important features of IBS, as demonstrated in rectal sensitivity experiments. Together with the observation of abnormal gut motility and alterations in enteroendocrine and neuropeptide systems in relation to the enteric and central nervous system as well as gut microbiota, perturbations in the gut-brain axis and its bidirectional communication have been proposed among the central mechanisms in IBS pathophysiology [5–7]. Other specific factors may instead be important only for specific subgroups of IBS patients, such as altered bile acid synthesis in IBS-D [5, 9].

## The genetic architecture of IBS

A heritable component of IBS has been demonstrated in twin and family studies, although heritability estimates have varied between 0 and 57 % [10]. Recently, however, a Swedish nationwide survey including more than 50,000 cases showed increased IBS risk among first-, second- and third-degree relatives, clearly indicating that a genetic component exists [11]. Evidence is now accumulating that genetic risk in IBS spans from complex polygenic conditions with combinations of common variants, to cases with rare single gene abnormalities [12]. For the majority of IBS patients, the genetic background will be constituted by a large set of common genetic variants, each contributing a small risk effect. At the same time, there may be subsets of patients where highly penetrant genetic variability in individual genes accounts for most of the phenotype. A good example of this phenomenon recently came from a study at the Mayo Clinic in collaboration with our group [13]: sequencing of the *SCN5A* gene in 584 IBS patients and 1380 asymptomatic controls identified functionally deleterious mutations in 2.2 % of IBS cases but none among controls. The *SCN5A* gene encodes the Na_V_1.5 ion channel responsible for the pacemaker function of the heart but is also present on interstitial cells of cajal, the ‘pacemaker’ cells of the gut. Most of the mutations identified were loss-of-function and carriers most often constipation-predominant. Of note, a severe IBS-C case with a highly penetrating *SCN5A* loss-of-function mutation could be successfully treated with mexiletine, a drug known to restore Na_V_1.5 channel function. In addition, also common *SCN5A* single nucleotide polymorphisms (SNPs) were found to affect IBS risk in our pilot genome-wide association study (GWAS) of ~5000 subjects (described further down), as well as in four independent case-control cohorts from Sweden, Italy, Greece, and the USA. These findings hence strengthen the hypothesis that both rare mutations and common variants may be implicated in IBS. However, overall, gene-hunting efforts in IBS have so far been scarce [12], and large-scale efforts remain to be carried out. Recognizing that IBS spans from complex polygenic conditions to rare single-gene forms, we need to adopt different strategies to identify these genetic factors.

## The challenge in finding IBS-risk genes: past and future perspectives

More than 60 candidate genes have been studied for IBS, including genes involved in serotonin synthesis and reuptake, mucosal immune activation and inflammation, neuropeptide signaling, nociception, bile acid synthesis, and intestinal secretion. These genetic findings have been reviewed and discussed thoroughly before, and a selection of genes showing best evidence of association to IBS is reported in Table 1 [10, 12]. However, all of these studies are candidate-gene case-control studies conducted in relatively small sample sets. Many of the identified associations have not been successfully replicated in independent studies, and although *p* values have been nominally significant, none of the findings are close to sustain the correction for a GWA study. Therefore, IBS-risk genes thus far proposed mostly still represent non-validated hits rather than true predisposing factors, and we have very few convincing IBS-risk genes identified so far. One exception may be the *TNFSF15* gene, which was convincingly associated in our original study [14] and then successfully replicated in independent cohorts [15–17]. In the original study, we hypothesized that genes involved in immune responses and host-microbe interactions may also contribute to IBS susceptibility, since these are mechanisms suggested to be involved in IBS pathophysiology [5, 7]. Therefore, we tested the association of polymorphisms (SNPs) from 30 known Crohn’s disease risk loci on IBS risk in two case-control studies from Sweden and the USA (total *n* = 1992). The G allele (risk allele for Crohn’s) of rs426839 in *TNFSF15* was significantly associated with increased risk of IBS (*p* = 2.2e-05; OR 1.37) and even more in IBS-C (*p* = 8.7e-07; OR 1.79). This gene encodes the TL1A protein expressed in immune cells, which promotes inflammatory response in the gut mucosa. Carrying the risk allele is suggested to result in higher TL1A expression and thereby stronger T cell activation and immune response. Hence, the association with *TNFS15* indicates that inflammatory response may be an important mechanism also in IBS.

**Table 1:** Prioritized IBS-risk genes based on existing published statistical evidence

Gene	Chr location	Gene name	Gene function	Gene region	Phenotype	Key reference
TNFSF15	9q32	Tumor necrosis factor (ligand) superfamily, member 15	Codes for TNF-like ligand 1A (TL1A), which contributes to the modulation of inflammatory responses.	Intron	IBS, IBS-C	[14]
				Intron and upstream	IBS-D	[15]
				Intron	IBS-A	[16]
				Intron	IBS, IBS-C	[17]
TLR9	3p21.3	Toll-like receptor 9	A Toll-like receptor that activate the immune system through recognition of specific patterns on microorganisms.	Intron and upstream	PI-IBS	[26]
				Upstream	IBS-D	[27]
HTR3E	3q27.1	5-hydroxytryptamine (serotonin) receptor 3E, ionotropic	A receptor for serotonin, a neurotransmitter in the central nervous system and gastrointestinal tract.	3′UTR	IBS-D	[28]
NPSR1	7p14.3	Neuropeptide S receptor 1	Receptor for neuropeptide S, expressed in brain and enteroendocrine cells, involved in anxiety, inflammation, nociception, etc.	Intron and coding polymorphism	Colonic transit time, sensory ratings (pain, gas, urgency)	[19]
				5′ near gene and beginning of gene	RAP	[21]
KLB	4p14	Klotho beta	Co-receptor of fibroblast growth factor 19 (FGFR4) on hepatocyte membrane, required for suppression of bile acid synthesis in liver.	Coding polymorphism	Colonic transit in IBS-D	[9]
SCN5A	3p21	Sodium channel, voltage gated, type V alpha subunit	Voltage-gated sodium channel (Na_V_1.5) in pacemaker cells of the heart and interstitial cells of cajal (ICC) cells of the gut important for smooth muscle contraction.	Rare coding mutations and signal of common SNPs in middle of gene	IBS, IBS-C	[13]
CDC42	1p36.1	Cell division cycle 42	A small GTPase of the Rho-subfamily involved in cell cycle regulation and possibly epithelial barrier function through intestinal stem cell differentiation and proliferation.	Intron	IBS-C	[16]
KDELR2	7p22.1	KDEL (Lys-Asp-Glu-Leu) endoplasmic reticulum protein retention receptor 2	Belongs to a family of KDEL motif binding receptors, mediating the retrograde transport of proteins to the endoplasmatic reticulum.	Intron	IBS	[25]

*Chr* chromosomal location, *IBS-D* diarrhea-predominant IBS, *IBS-C* constipation-predominant IBS, *IBS-A* alternating IBS, *PI-IBS* post-infectious IBS, *RAP* recurrent abdominal pain

An alternative approach to the discovery of IBS relevant genes is to study the endophenotypes, otherwise called intermediate phenotypes of disease. These are usually quantitative traits related to IBS, such as colonic transit time and visceral sensitivity ratings. To focus on biological observations instead of clinical entities for establishing, diagnosis was proposed already 50 years ago, and this alternative strategy has been used successfully for genetic studies in the psychiatric field where illnesses often are, as IBS, complex and heterogeneous [18]. The concept of endophenotypes is to use disease-associated phenotypes in an attempt to reduce the complexity and to increase chances of finding genes important to biological processes underlying disease etiology. For the case of IBS, suitable traits to use for this approach include bowel movement frequency, Bristol stool form scale (a subjective measure of stool consistency, related to colonic transit time), pain and sensation ratings in response to visceral stimuli, and others. Previous work from our group has shown associations between polymorphisms in the neuropeptide S receptor gene (*NPSR1*) and colonic transit time, as well as gastrointestinal (GI) sensory ratings, such as gas, pain, and urgency [19]. The NPSR1 protein is a receptor for neuropeptide S, a neuropeptide involved in anxiety, response to stress and fear, inflammation, and nociception. Neuropeptides act in the brain-gut axis and have been implicated in IBS before [2, 6]. We also showed that the neuropeptide S (NPS)-NPSR1 system can induce the expression of other neuropeptides in vitro [19, 20]*.* Hence, this gene was a plausible candidate to investigate also for its potential genetic associations with abdominal pain. In the Swedish BAMSE birth cohort, we performed a candidate-gene study in relation to recurrent abdominal pain (RAP) [21], which occurs frequently among children and is one of the cardinal symptoms of FGIDs. The mechanisms of visceral pain and RAP are not fully understood, a heritable component has been demonstrated, and a few candidate genes proposed. We observed association with RAP at 7/24 tested SNPs, with the strongest signal in correspondence of a putative regulatory region upstream *NPSR1* where they may exert their genetic effects through the modulation of gene expression.

Overall, IBS poses some major challenges, and it appears that in order to overcome these and be able to identify true unequivocal risk genes and variants, we need to implement larger scale analyses. Genetic-risk genes and variants have been successfully identified and replicated in a plethora of complex diseases using the powerful and hypothesis-free approach of GWAS studies and their meta-analyses (see the NHGRI-EBI GWAS Catalog; www.ebi.ac.uk/gwas) [22]. For instance in the GI field, a recent study reported the identification of 38 additional susceptibility loci for inflammatory bowel disease (IBD), bringing the total tally of confirmed IBD risk loci to 200 [23]. On the contrary, IBS genetics is very much lagging behind, and no similar GWAS effort had been attempted before our Screening-Across Life Span Twin (SALT) study (see below).

GWA studies are powerful approaches to discovering genetic risk loci in complex diseases [24], though this has never even been attempted in IBS. In order to reach the statistical power necessary to detect meaningful association, very large sample size is required. Unfortunately, this is currently unfeasible in the IBS community, as the number and size of well-characterized cohorts around the world is still surprisingly slim. In fact, only a portion of individuals suffering from IBS according to the Rome III criteria seek medical attention for their symptoms, with numbers reported ranging from 10 to 70 % [1]. In order to reach more individuals, we propose to shift approach and instead make use of general population samples for discovery purposes in IBS genetics. Large biobanks and general population cohorts offer a great opportunity to perform genetic epidemiological association studies with considerably larger sample sizes. When available, Rome questionnaire data may be used to identify IBS cases and asymptomatic controls in these large study populations. In addition, genotype data may also be linked to phenotype in these studies using the International Classification of Diseases (ICD codes) and electronic medical records (EMR). By shifting to a general population approach for the discovery phase, we gain considerable sample size and improve in the definition of controls, as they are co-sampled with the cases and classified with the very same investigative tools (for example questionnaires). However, once IBS-risk genes and variants have been discovered, validation of results should be performed by replication and targeted analyses in case-control cohorts from gastro clinics with well-characterized IBS patients and healthy controls. Furthermore, investigating rare variants/mutations in IBS, next-generation sequencing in selected cases and controls will also be necessary.

Our group recently conducted the very first pilot GWAS of IBS [25]. In this study, Rome II criteria from questionnaires were used to identify 534 IBS cases and 4932 asymptomatic controls from the SALT study of the Swedish Twin Registry. A GWAS was performed and top regions replicated in six independent case-control studies with patients and controls recruited from European and US clinics. Confirmatory, one of the loci (harboring the *KDELR2* gene) was successfully replicated in all these samples (total sample size of 8977) with consistent effect of association. Even though sample size was still too small to reach genome-wide significance, this study corroborated our hypothesis that general population-based cohorts with associated genetic and epidemiological/healthcare data provide excellent opportunities to study the genetic architecture of IBS and related GI symptoms.

Overall, the ultimate value of genetic research in IBS is to identify key physiological mechanisms, which will help us understand its pathophysiology. These findings will help improve IBS diagnosis and classification and therefore impact therapeutic strategies and personalized treatments.

## Abbreviations

FGID: functional gastrointestinal disorder
GI: gastrointestinal
GWAS: genome-wide association study
IBS: irritable bowel syndrome
IBS-C: constipation-predominant IBS
IBS-D: diarrhea-predominant IBS
IBS-M: mixed IBS phenotyped
